# Chasing *Pinna nobilis* Survivors: Current Status in Spanish Open Coastal Waters

**DOI:** 10.3390/ani15213075

**Published:** 2025-10-23

**Authors:** Francesco Maresca, Elvira Álvarez, Lara Zafra, Iris E. Hendriks, Gaetano Catanese, Raul González, José Rafael García-March, Maite Vázquez-Luis

**Affiliations:** 1 Instituto Español de Oceanografía-C.O. Baleares (IEO, CSIC), 07015 Palma de Mallorca, Spain; elvira.alvarez@ieo.csic.es (E.Á.); lzafrapr@gmail.com (L.Z.); raul.gonzalez@ieo.csic.es (R.G.); 2 Oceanography and Global Change Department, Mediterranean Institute for Advanced Studies (CSIC-UIB), 07190 Esporles, Spain; iris@imedea.uib-csic.es; 3 IRFAP-LIMIA Laboratorio de Investigaciones Marinas y Acuicultura-Govern de les Illes Balears, 07157 Port d’Andratx, Spain; gaetano.catanese@uib.es; 4 INAGEA (UIB)-Instituto de Investigaciones Agroambientales y de Economía del Agua, Universidad de las Islas Baleares, 07122 Palma de Mallorca, Spain; 5 Instituto de Investigación en Medio Ambiente y Ciencia Marina (IMEDMAR-UCV), Universidad Católica de Valencia SVM, Calpe, 03710 Alicante, Spain; jr.garcia@ucv.es

**Keywords:** critically endangered, endemic, monitoring, bivalve, mass mortality event, citizen science

## Abstract

**Simple Summary:**

The largest and endemic bivalve of the Mediterranean Sea, *Pinna nobilis*, is on the brink of extinction after a mass mortality event that has affected its populations since autumn 2016. In the attempt to recover these populations, several measures have been proposed by an international group of experts. Among these measures, the search and monitoring of surviving individuals in affected areas are crucial to understanding the evolution of the mortality event. This study provides the knowledge acquired from an 8-year period of monitoring the surviving individuals of *P. nobilis*. The results located the hotspots of the presence of survivors and revealed that an increasing proportion of these individuals were hybrids (between *P. nobilis* and *P. rudis*). In some cases, these individuals were threatened by human activities (i.e., anchoring, fishing), which should be regulated to avoid additional pressures other than the mass mortality event. Furthermore, adequately performed translocation appeared to be a good measure to dispel the imminent threat to the existing surviving individuals. Additionally, almost half of the records of surviving individuals included in this study were provided by a non-scientific community, highlighting the importance of citizen science in the conservation of an emblematic species on the brink of extinction.

**Abstract:**

The largest and endemic bivalve of the Mediterranean Sea, *Pinna nobilis*, is on the brink of extinction after a mass mortality event (MME) that has affected its populations since autumn 2016. Since then, different actions have been performed to improve the conservation status of *P. nobilis*. The monitoring of survivors in open coastal systems along the Spanish Mediterranean coast showed, after an 8-year period since the start of the MME (2017–2024), that the geographical distribution of the survivors in open sea is currently concentrated in a few regions, with focal points of specimen density in Cap de Creus (Catalonia) and Menorca (Balearic Islands). During the exhaustive monitoring of individuals of *P. nobilis*, the active participation of citizen science became decisive, locating almost half of the survivors. Most individuals were found in marine protected areas, mainly in *Posidonia oceanica* meadows in the upper 15 m. As a safety measure, several survivors were translocated to safer areas, while evaluation of the impact of the translocation showed no demonstrable effects. The knowledge acquired during these years has highlighted the necessity for collaborative monitoring, specifically to understand the current critical situation of *P. nobilis* and to implement effective conservation measures for this emblematic species.

## 1. Introduction

Mass mortality events (MMEs) are demographic catastrophes that can rapidly remove a substantial proportion of a population, regardless of life stages, over a relatively short period of time [1]. These events are often associated with bacterial and/or fungal disease, human perturbations, biotoxicity, climatic factors (weather, thermal stress, starvation) and the interactions between these drivers [1]. As a result, MMEs can result in local population extinction, driven by the loss of genetic diversity, demographic stochasticity and the Allee effect [2]. The most severe MMEs seem to be caused by an interaction of stressors, starvation and disease, while it is uncertain if the reported increased frequency of MMEs for marine invertebrates is real or is due to an augmented perception of such events [1].

In this context, an MME affecting the bivalve *Pinna nobilis* (Linnaeus, 1758) started in the western Mediterranean Sea in early autumn 2016 [3]. Initially, this MME appeared to be associated with a new species of haplosporid parasite [3,4] named *Haplosporidium pinnae*, which was considered to specifically affect *P. nobilis* [5]. However, more recent findings suggested the presence of this parasite in other hosts before the MME [6]. Although *H. pinnae* was identified as the main pathogen associated with the MME and the only common agent detected across all Mediterranean areas [7], other studies suggest that mortality could, in different contexts, be a consequence of co-infection or multifactorial disease involving *Vibrio mediterranei*, *Mycobacterium* sp., *Perkinsus* spp. and picornaviruses [6,8,9,10,11,12,13]. As a consequence, this event led to a drastic population decline close to 100% across its range in open coastal areas in the Mediterranean Sea [14], affecting specimens of all sizes, depth ranges and habitat types.

The fan mussel *P. nobilis* is the largest endemic bivalve in the Mediterranean Sea [15], and can live up to 50 years [16], depending on the quality of the environmental conditions [17]. It lives partially buried in the substrate, at a depth range of 0.5 to 60 m, in a variety of habitats, like sand, detritus and meadows of *Cymodocea nodosa*, *Zostera marina* and *Zostera noltii*, even though its preferred habitat is *Posidonia oceanica* meadows [18,19,20,21]. *P. nobilis* plays an important ecological role: it contributes to water clarity [22] through its capacity to filter more than 2000 L of seawater per day [23], and it provides hard structures in soft substrate habitats, hence adding structural complexity and species diversity to the ecosystem since multiple benthic species can attach to its valves [24,25,26,27].

Aside from the ecological functions it provides, *P. nobilis* has also been considered an emblematic species, used as an ornament and in textile manufacturing since the Babylonian period [28]. During the last decades, fan mussel populations have significantly declined due to anthropogenic activities [29], such as excessive exploitation [21,30], habitat loss [31], fishing, anchoring, diving [32,33,34] and pollution [29,35].

To cope with these multiple stressors affecting this culturally and ecologically important species, *P. nobilis* has been granted strict protection under Annex II of the Barcelona Convention, listed as “Vulnerable” (SPA/BD Protocol, 1995). Successively, as a consequence of the MME, the status of this species in Spain has changed from “Vulnerable” to “Critically Endangered” [36,37], and conservation strategies have been developed at national [38] and international [39] levels, while the species is also included in the IUCN Red List [40]. In response to the MME, the scientific community has undertaken several initiatives aiding the conservation of the fan mussel [41], such as the monitoring of unimpacted populations [42,43], captive breeding [22,23] and, under certain circumstances, translocation of specimens (i.e., from areas with high frequency of human activity into a marine protected area), which is considered a quite effective conservation measure [44,45,46].

During the search for surviving individuals in the Mediterranean Sea, a few resistant individuals were encountered in open coastal areas across the Mediterranean [3,47]. Some of the specimens proved to be the result of natural hybridization between *P. nobilis* and *P. rudis*, its closest relative [48]. The factors involved in the survival of these isolated individuals remain poorly known, yet they may be crucial for the conservation and restoration of the species. However, despite the collaborative efforts by the scientific community to locate surviving individuals, a comprehensive study of these individuals, including data on their spatial and temporal distribution, is still lacking.

In recent years, the application of citizen science in marine conservation has increased for a variety of topics [49,50,51], including endangered species monitoring [52]. One of the reasons behind this success is that this approach provides data at large spatial and temporal scales [53,54,55] at a relatively low cost [56]. At the same time, citizen science does have an impact on the democratization of science and the involvement of citizens in the management and conservation of the natural environment [57,58,59]. It is important to note that the scientific literature highlights important caveats regarding the application of citizen science in ecological research, such as the reliability of data obtained through this methodology [60,61], which needs to be properly addressed to achieve the standards required by the scientific community and policy makers [60,61,62].

Therefore, the present manuscript aims to implement a comprehensive study of the survival of *P. nobilis* individuals in open coastal areas after the MME. Specifically, the objectives are (i) to explore the spatial and temporal patterns of persisting individuals, (ii) to perform a pilot translocation study of survivors and (iii) to clarify the taxonomic status of the encountered individuals after the MME (pure *P. nobilis* vs. hybrids).

## 2. Materials and Methods

### 2.1. Study Area and Data Collection

**Figure A1:**
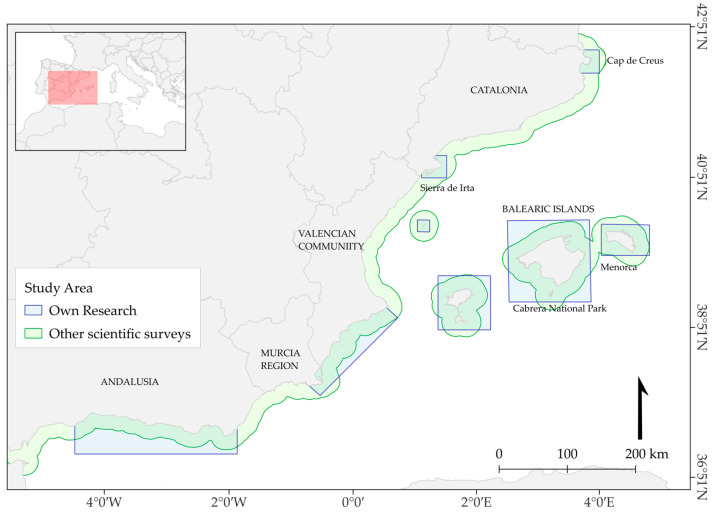
Map of the study area along the Mediterranean coast of Spain. The red box indicates the enlarged area. Blue areas show where surveys and monitoring have been carried out by the scientific team, and the green areas show where citizen science has been conducted. Arrow indicates the north.

The present study covered a monitoring period spanning just over seven years, from March 2017 to December 2024. During this period, individuals of *P. nobilis* along the Spanish Mediterranean coast were monitored, with the participation of citizens, technicians and scientists in the acquisition of data. To obtain data of surviving individuals over a large spatial scale, the research team (IEO-CSIC) first performed 20 specific field surveys in the Balearic Islands, Catalonia, the Valencian Community and the region of Murcia. The search for surviving individuals was carried out along the coast of the Levantine–Balearic marine demarcation, in vegetated areas with *P. oceanica* and *Cymodocea nodosa*, as well as on sandy bottoms. The study area includes marine areas subject to various levels of protection, such as National Parks, Marine Reserves, Natural Parks or Sites of Community Importance and/or Special Areas of Conservation of the European Union Nature 2000 Network (SCI/SAC). During these surveys, a diver propulsion vehicle (DPV) was used to perform several underwater transects in suitable habitats to spot, geolocate and evaluate the state of any encountered living specimen according to the methodology of [3]. Additionally, the geographical scale of the exploration was increased by a collaborative effort between governmental institutions, different research groups involved in the study of the species and the use of a citizen science network (see Figure A1).

The public was successfully involved through collaboration with dedicated platforms (i.e., “Sea Watchers (SW)” http://www.observadoresdelmar.es/; accesed on 15/01/2024), and local platforms such as the Species Protection Service of the Balearic Islands regional government) and also recruited through dissemination campaigns to spread awareness on the current state of the fan mussel and to encourage the participation of citizens to report any spotted surviving specimen. Among all the notifications of *P. nobilis* survivors, only those containing photos or videos that allowed for the identification of the species were considered, as the presence of living hybrids between *P. nobilis* x *P. rudis* (hybrids hereafter) was also recorded. Once the information obtained from the citizen science platforms was validated, the surviving individuals were visited to ensure compatible data with the visual censuses: each specimen was taxonomically identified (*P. nobilis* vs. hybrid; for further details, see [48]), photographed, tagged, geolocated, and measured. Their health status and the existing environmental pressures present at their respective locations (anchoring, fishing nets, wastewater discharge, etc.) were also recorded. After the first registration, survivors were monitored periodically.

When an individual was considered threatened by nearby pressures, relevant authorities were informed in order to request its translocation to a safer location. If granted, the procedure was performed in collaboration with local managers according to previously developed and tested protocols [47,63], highlighting the importance of conserving the structural integrity of the byssus, a crucial factor for its successful reintroduction into the environment.

Age at death of ten individuals was estimated from the posterior adductor muscle scars (PAMSs) recorded from their empty shells. These shells were considered a representation of the entire group of survivors because they had similar size and shape to the rest. Aging was based on the methodology proposed by [17,64].

### 2.2. Molecular Analyses

Surviving individuals were biopsied, whenever possible, during the monitoring visits. This procedure was conducted using a non-lethal sampling method described in [65,66], consisting of the collection of a small portion of the mantle tissue from an individual using tweezers. The samples obtained were immediately immersed in 100% ethanol and stored at room temperature after returning from the immersion. The sampling was carried out under strict compliance with the permits granted by the competent authorities, given its status as an endangered and protected species (Ministry for the Ecological Transition: SGPM/BDM/AUTSPP/69/2019, SGBTM/BDM/AUTSPP/12/2024; in the Balearic Islands: CEP 18/2017, CEP 17/2019, CEP 23/2022, ESP 15/2023, ESP 13/2024, SEN 885/202, SRM 26/2023 ERM, SEN 433/17, SEN 54/18, SEN 100/19, SEN 38/20, SEN 376/23, SEN 353/24; in Catalonia: SF/0709, SF/0086/22, SF/0003/23, SF0143/24, SGYB/JEM, FUE-2022-1560931, FUE-2024-03814876, 2018PNATCCRAUT0062, FUE-2022-02509114, FUE-2022-02918037, FUE-2024-04254621; in the Valencian Community: NRE_692, AUT 04/22; in the region of Murcia: 2019_0676_AC3_MEN_ATZ, 2020_0301_AC3_LIT_ATZ, ENP20230264_AC3_BIS; and in Andalusia: SGYB/JEM, PNCGN_24/24_305, AUT 02/24, SENP/MNCS).

Total genomic DNA was extracted from mantle tissue samples using the DNA NucleoSpin^®^ Tissue extraction kit (Macherey-Nagel, Duren, Germany) following the manufacturer’s instructions. The extracted DNAs were thoroughly analyzed in the laboratory to achieve two primary objectives. One objective focused on confirming the taxonomic status of the specimens by genetically discriminating between *P. nobilis* and hybrid individuals. To achieve this, two complementary molecular approaches were utilized. The first involved a non-destructive, rapid identification method based on characteristic differences in mitochondrial genome sequences [67]. This method, originally developed for juvenile identification using environmental DNA (eDNA) samples to minimize sample damage [68], was adapted in this study for use with tissue samples.

The second approach employed a multiplex PCR assay targeting nuclear DNA markers, providing a robust means to distinguish pure species and hybrids by amplifying species-specific nuclear loci [69]. All the PCR conditions used in this work were the same as described by the cited authors. The PCR products were separated on 2% agarose gels in TAE 1x buffer (*w*/*v*). The gels were stained with GelRed and included a HighRanger DNA ladder size standard of 50 bp (for mitochondrial PCR) or 1 kb (for nuclear PCR amplification).

The other major objective was the evaluation of the physiological condition of the specimens, with particular emphasis on screening for potential pathogenic infections. Diagnostic assays were applied to detect the presence of *Mycobacterium* spp. and *H. pinnae.* Primers HPNF3/HPNR3 [5] and HpF3/HpR3 [70] were used for detection of *H. pinnae*. Moreover, the primers mycgen-f/mycgen-r, described by [71], were utilized to detect the presence of *Mycobacterium* sp. in the same samples. The PCR conditions reported in the cited articles were also applied in these cases.

### 2.3. Data Structure and Analyses

The records of surviving individuals of *P. nobilis* or hybrids can be grouped according to five sources (scuba diver, diving center, scientist/technician, fisherman, volunteer). Similarly, all the information channels used to notify the records were grouped into six categories (own data, direct contact, SW, press, social networks and the wildlife protection service).

Among all the reported and validated individuals, only surviving individuals are included in this study. These were defined as *P. nobilis* or hybrid individuals that overcame the local onset of the MME and were found alive after mortality in their area had reached nearly 100%. It should be noted that the MME did not start at the same time in all locations; in fact, the first and last areas affected by the MME in the Spanish NW Mediterranean Sea were almost 2 years apart, at the end of September 2016 and June 2018, respectively [3,47,72,73]. The dates of the first local detection of the MME reported in these publications were considered in this study.

Despite the regular monitoring, there were instances where the exact moment of death, hence the survival time, could not be determined. Therefore, to evaluate the survival patterns of these individuals, the monitoring time was used as a proxy for the survival time, since it was a metric available for all of the specimens. The monitoring time alive (TMA) was defined as the period between the first report of a surviving individual and the moment when it was seen or declared dead. In some instances, the specimens could not be located; in this case, they were declared dead when the specimen was still missing after two consecutive monitoring visits.

Based on an in situ evaluation, satellite imagery, local regulations and existing georeferenced datasets of diving sites (Professional Association of Diving Instructors—PADI) and wastewater output (EMODnet dataset), the occurrence of human activities that could potentially affect the surviving individuals was established. Human activities considered as pressures for *P. nobilis* were anchoring, fishing, SCUBA diving, other recreational activities (swimming) and sewage discharge [33]. Additionally, the effect of marine protected areas was explored by assigning a score to each surviving individual, depending on where it was located. This score depended on the existing protection measures, from strongest to weakest, according to the national legislation [33]: National Parks (1), Marine Reserves (2), Natural Parks (3), “SCI/SAC” sites (4) and No protection (5).

Regarding translocations, it is important to highlight that, in the past, many failures in translocation procedures have occurred [74,75]. To resolve these issues, meticulous attention was given to ensure the effectiveness of these interventions. The criteria used to boost the translocation success included careful evaluation and selection of the recipient habitat to ensure its suitability for the translocated species, which is a fundamental aspect in translocation projects [76,77]. In addition, special attention was also provided to the methodology of the translocations, following the protocols established in the scientific literature [63], as well as to the monitoring of the translocated individuals [75,76,78,79]. Thus, to investigate the effect of translocation on the survival of the specimens, the mean TMA, starting from the translocation event, was compared between translocated (n = 7) and non-translocated (n = 27) specimens. The non-translocated specimens were selected using the criteria that they were first encountered on a similar date and in an area close to the translocated ones.

The effect of different factors (hybridization, biopsy and translocation) on the TMA (and, thus, survival) was tested with a Mann–Whitney U test. Additionally, the Kruskal–Wallis test (with Bonferroni correction) was performed to explore the difference in the same variable between the 5 protection levels. In this case, pairwise comparisons were performed with Dunn’s test. All analyses and figures were performed and created with R software (version 4.4.2).

## 3. Results

During the monitoring period, a total of 153 individuals were found alive after the occurrence of the MME in the respective localities, with a total of 531 visits/observations (including both first sightings and repeated monitoring visits) of *P. nobilis* and hybrids recorded by the authors throughout the study, with the highest frequency of visits occurring in 2018 (151 visits, 28.4% of total visits) and the lowest in 2021 (17 visits, 3.2%). Similarly, the initial two-year monitoring period was characterized by the highest number of monitored individuals (62 and 71, respectively). In the following years, the number of monitored individuals decreased (21.5 ± 2.4 individuals per year), with the minimum number of individuals (15) observed in 2021. It is worth noting that the monitoring effort decreased during 2020 and 2021, due to the COVID-19 pandemic, but it slightly increased again during 2022–2024 (8.3%, 16.0% and 9.2%, respectively).

**Figure A2:**
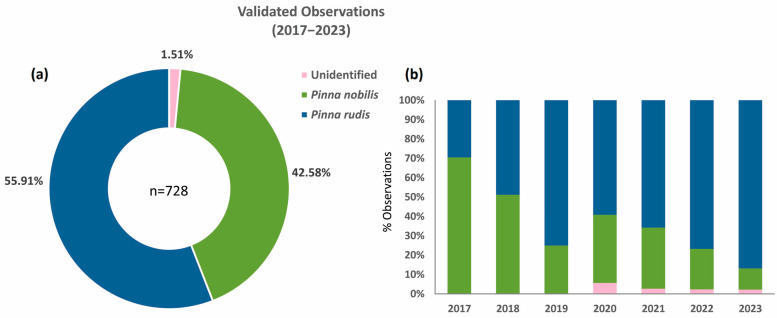
The NACRAS project of the SW citizen platform received 728 observations of fan mussels during this period, validated by the research team through the photographs provided by the observers. More than half of the total number of these observations corresponded to *Pinna rudis* (55.91%), whereas 42.58% of the observations corresponded to *Pinna nobilis.* Species identification was not possible in 1.51% of the reports. The number of *P. nobilis* observations decreased with the expansion of the MME, from 70.5% of observations in 2017 to only 11% in 2023. At the same time, the number of *P. rudis* observations increased over the years and, in 2023, was higher than 85% of the observations reported in that year. It is also worth noting that not all *P. nobilis* observations later validated on the platform corresponded to surviving specimens. (a) Percentage of total observations of the Sea Watchers platform regarding each species and (b) annual percentage of total observations of the Sea Watchers platform per year.

During the same period, the citizen science platform SW generated a total of 728 observations of fan mussels through the NACRAS module. In total, 42.58% of these observations corresponded to *P. nobilis* specimens. However, the annual proportion of *P. nobilis* observations decreased with the expansion of the MME. In fact, the proportion of *P. nobilis* observations obtained by the platform ranged between 70.5% of the total number of observations and only 11%, obtained in 2017 and 2023, respectively (see Figure A2). However, among all of the 310 validated *P. nobilis* observations on the platform, only 14 corresponded to specimens surviving the MME.

Considering only the specimens of *P. nobilis* and hybrids that were found alive after the local onset of the MME and at any time along the monitoring period (2017–2024) in the Spanish Mediterranean coast from the aforementioned data sources, a total of 153 fan mussels constituted the dataset used for this study. Among these surviving fan mussels, 134 were identified as *P. nobilis*, whereas 19 were determined to be hybrids, with their proportion increasing each year (Table 1).

**Figure 1:**
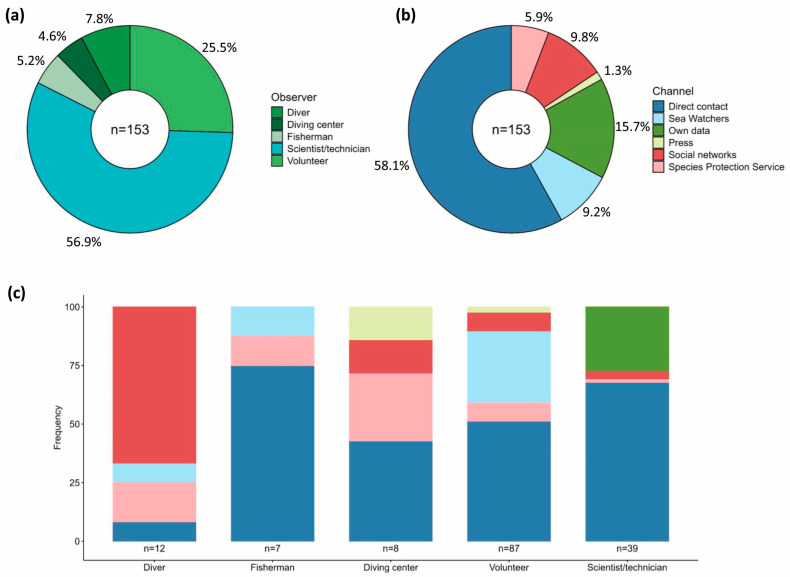
Observations of surviving *P. nobilis* and hybrids during the monitoring period (2017–2024). (**a**) Source of the collected observations of survivors. (**b**) Information channels used to transmit observations of surviving specimens to the COB-IEO-CSIC team. (**c**) Proportion of most used information channels by source category, colors codes are the same than (**b**).

Most of the observations were made by scientific and technical personnel (56.9%), while the rest was contributed through citizen science profiles, including volunteer observers (25.5%), autonomous scuba divers (7.8%), dive centers (4.6%) and underwater fishermen (5.2%) (Figure 1a).

These observations were reported through different channels, of which direct communication with researchers, either by email or telephone, was predominant (58.2% of cases), followed by the scientific monitoring program (15.7%), social media (9.8%), the citizen science platform SW (9.2%), reports via the Species Protection Service (SPS) (5.9%) and, finally, through press publications (1.3%) (Figure 1b). Direct reporting is the predominant channel for persons adhering to the scientific–technical profile (66.3% of cases), although it is also used by the rest of the observer profiles, except for scuba divers. This last profile differs most in communication style, as 72.7% of the sightings are posted on social media (Figure 1c).

**Figure 2:**
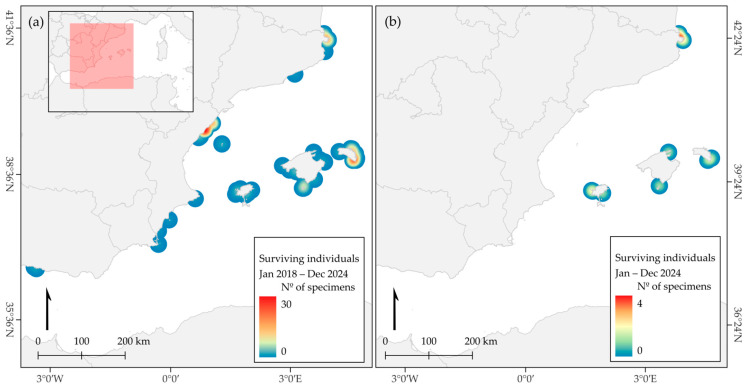
Heat map generated with Kernel density estimation (20 km search radius): (**a**) for the cumulative total of *P. nobilis* and hybrid survivors (n = 153) during the study period 2017–2024, and (**b**) for survivors in December 2024 (n = 12). The red box indicates the enlarged area. Note that scale bars among figures differ, arrow indicates the north.

The highest number of survivors was located in the Balearic Islands (44.4%), the Valencian Community (33.9%) and Catalonia (15.7%). Four areas showed a particularly high density: Sierra de Irta and Prat de Cabanes in the Valencian region, where 44 specimens of *P. nobilis* were identified; Cap de Creus in Catalonia, with 22 recorded specimens of *P. nobilis*; the northeast coast of Menorca in the Balearic Islands, where 41 individuals (38 *P. nobilis* and 3 hybrids) were monitored; and the Cabrera National Park, which hosted 7 individuals (4 *P. nobilis* and 3 hybrids) (Figure 2a). Over time, the presence of specimens disappeared in some regions, while it persisted in others throughout the study period, as shown in the distribution of survivors at the end of 2024 (Figure 2b).

**Figure 3:**
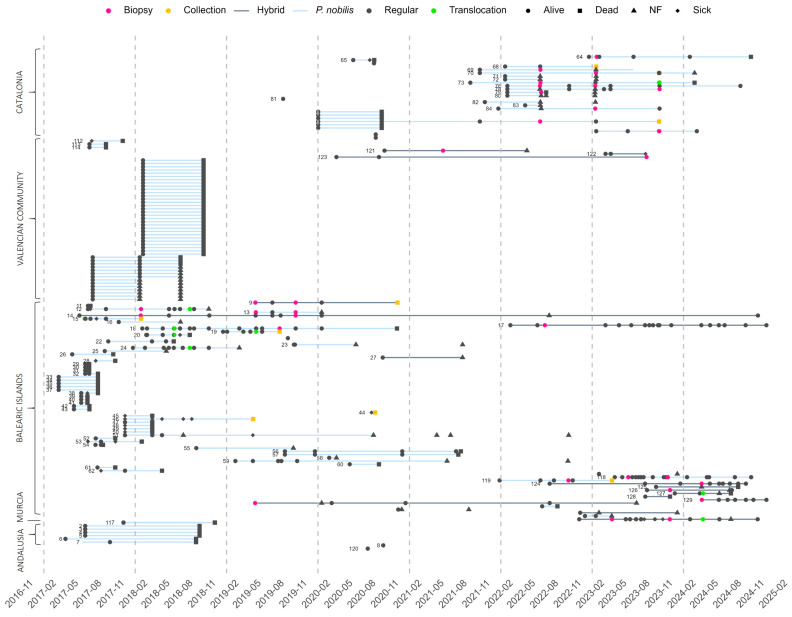
Temporal monitoring of *P. nobilis* and hybrid individuals. Segments show the monitored time alive for each individual, with blue for *P. nobilis* and black for hybrids. Visits are represented by forms, varying in color according to observation.

Initially, most survivors were detected in the Valencian region, where a significant number (47) were recorded between 2017 and 2018; however, all of them died before the end of 2018. A similar fate affected six individuals in Andalusia and one in the region of Murcia, where no further survivors were detected in the following years. Conversely, in the remaining two regions, Catalonia and the Balearic Islands, in particular Cap de Creus and Menorca, the appearance of new survivors never stopped during the study period. By December 2024, only two regions—Cap de Creus and the Balearic Islands—retained surviving individuals, accounting for 41.7% and 58.3% of the remaining specimens (12), respectively. The Balearic Islands also stand out for hosting specimens with the longest TMA and the highest concentration of hybrid individuals, either dead or alive (14; 73.7% of the total number of hybrids) (Figure 3). This region currently has the highest number of living individuals (7), of which only one is *P. nobilis*. In the northernmost region, Catalonia, a total of 24 individuals were monitored between 2020 and 2022, and there are currently 5 living individuals, all of them pure *P. nobilis*. Of the 12 living individuals monitored, 6 are identified as hybrids (50.0%), while the remaining 6 are classified as *P. nobilis* (50.0%).

The majority of the individuals (92.8%) were found in *P. oceanica* meadows, at depths ranging between 1 and 33 m, with 84.3% located in the upper 15 m. In total, 64.1% of individuals were found in regions that had some form of protection, with 4.6% located in National Parks, 20.3% in Marine Reserves, 9.8% in Natural Parks and 29.4% in lower-level protection areas, including “Nature 2000” network sites (SCI/SAC). The remaining 35.9% of the surviving specimens were found in unprotected areas. There were 12 individuals alive at the end of 2024, and 7 individuals that had only one observation until December 2024. These seven individuals were not taken into account for the analyses related to the monitoring period. Therefore, the remaining 134 individuals were monitored during the period from 03/2017 to 12/2024 (Figure 3). Of the 10 aged shells, most of them were young individuals, spanning from 3 to 15 years old (9 were younger than 10 years, and only one spanned to 15 years old) (Table 2).

**Figure 4:**
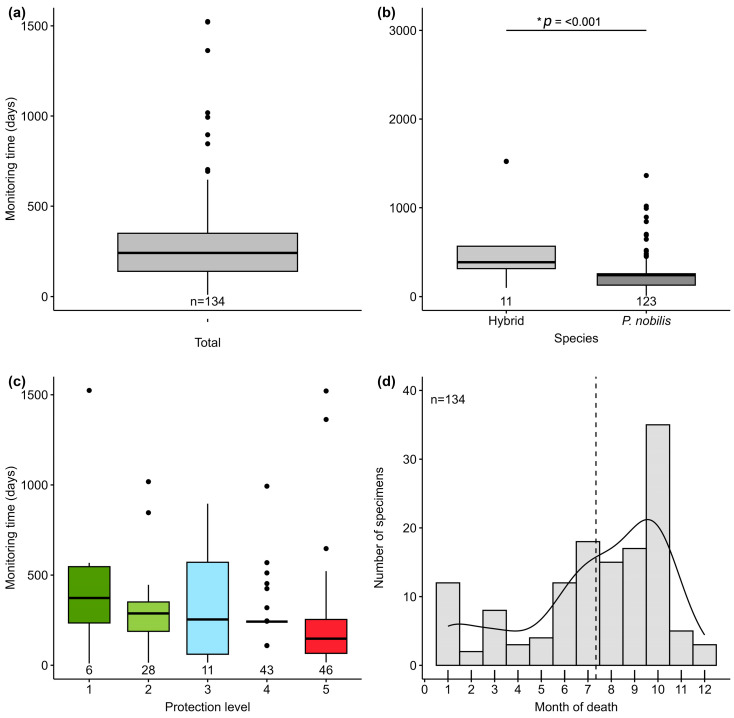
Monitoring time of surviving *P. nobilis* and hybrids by total number of surviving specimens: (**a**) both *P. nobilis* and hybrids, (**b**) by taxonomic identity and (**c**) according to the protection status of the marine area they inhabit: National Park (1), Marine Reserves (2), Natural Parks (3), SCI/SAC sites (4) and unprotected areas (5). (**d**) Monthly reported deaths accumulated over all the years studied (2017–2024). Black line with * indicates significant difference between groups. The dots are outliers and dotted line the mean.

Of all monitored individuals, 94.8% were reported dead within the first 2 years of detection, with an average TMA after detection of 283.43 ± 22.51 days (Figure 4a). *P. nobilis* and hybrids had different survival rates (W = 696, *p* = 0.03, n = 134), with hybrids surviving longer (572.6 ± 147.5 days) compared to *P. nobilis* individuals (257.5 ± 19.4 days) (Figure 4b). Protection status did not affect the survival time (Χ^2^ = 6.24, *p* = 0.182, df = 4). The specimen with the highest TMA in a National Park was 1524 days and was still alive at the moment of the MS writing, with an average of 310.00 ± 99.0 days of survival in a National Park, while the lowest TMA was found in unprotected areas (249.0 ± 47.6 days) (Figure 4c). Mortality was clearly seasonal, as 72.39% of deaths occurred between June and October, with the highest cumulative mortality rate in October, since 26.1% of dead individuals (n = 35) were found in this month (Figure 4d).

**Figure 5:**
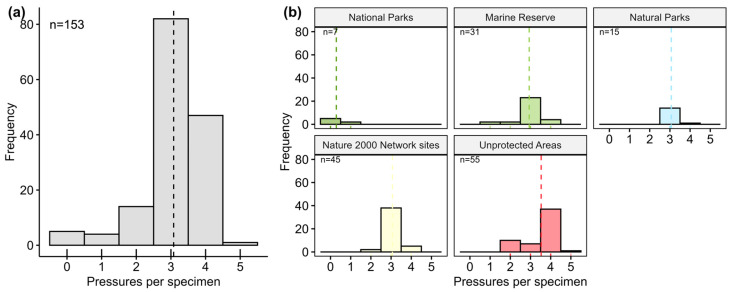
Frequency of surviving fan mussels according to simultaneously observed anthropogenic pressures: (**a**) globally and (**b**) by protection level. The dotted line is the mean. Colors represent the protection status of the marine area they inhabit: National Park (dark green), Marine Reserves (green), Natural Parks (blue), SCI/SAC sites (yellow) and unprotected areas (red).

Surviving individuals faced multiple anthropogenic pressures, with 94.1% exposed to more than one stressor, and 53.59% of them affected by three pressures simultaneously (Figure 5a). The most prevalent pressures were fishing (31.21%), anchoring (23.99%), recreational swimmers (19.32%), wastewater discharge (18.68%) and scuba diving (6.79%). Individuals in unprotected areas were subject to the highest number of simultaneous pressures described (4 or 5), whereas those in National Parks were affected by the least number of pressures. The majority of survivors were subject to three anthropogenic pressures, and most of them were located in SCI/SAC sites and Marine Reserves. It is worth noting that in the case of Marine Reserves and Natural Parks, the survivors were located outside of no-take areas (Figure 5b).

During the monitoring period, a total of nine *P. nobilis* specimens and hybrids were translocated from their original location to a more suitable location to improve their chances of survival. In most cases the specimens were threatened by anthropogenic impacts, and in two cases, the translocation was carried out after the specimen had been damaged by physical impact (specimens C^a^ and G^c^). Most of the translocations were carried out on specimens from the Balearic Islands (6), while one of them was from Cap de Creus (Catalonia) (G^c^) (Table 3). The destination in the Balearic Islands was the Cabrera National Park (6), since it has one of the most suitable conditions for the species (*P. oceanica* meadows), and has, historically, hosted dense populations of *P. nobilis* [20] before the MME. It is an area protected from anthropogenic impacts (no-take area), with excellent indicators of demography (mortality rates, recruitment rate and population growth rate) prior to the MME [80] and oceanographic connectivity [81].

The specimen from Cap de Creus (G^c^) was not pinpointed for translocation, but as the specimen was found displaced and severely damaged by physical impact during a survey, it had to be moved urgently. In this case, the destination location (Arenella islet) also harbors a preferred habitat and is protected from most physical impacts since it is a transit area. Unfortunately, the specimen was found dead in the last survey in February 2024.

**Figure 6:**
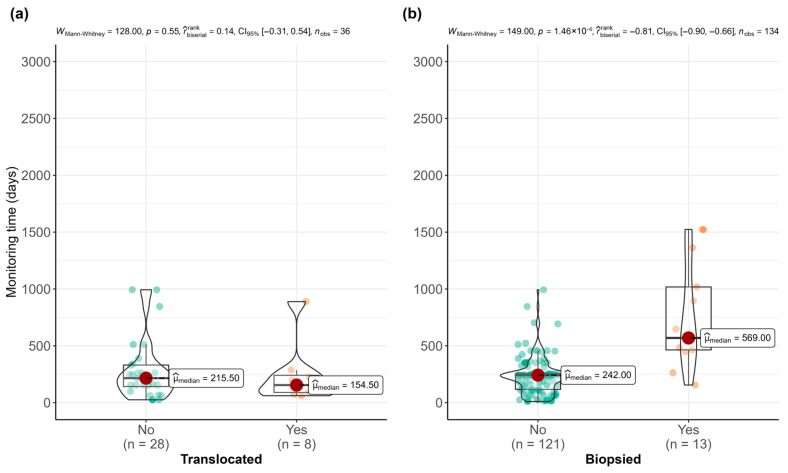
Mann–Whitney U test results comparing monitoring time between (**a**) translocated (n = 7) and non-translocated (n = 27) *P. nobilis* and hybrids, and (**b**) biopsied (n = 13) and non-biopsied (n = 121) *P. nobilis* and hybrids. Green and orange dots indicate “No” and “Yes” individuals respectively.

The monitoring (survival) time of translocated and non-translocated individuals was similar (W_Mann–Whitney_ = 41.0, *p* = 0.970) (Figure 6a). Over time, almost all translocated and non-translocated specimens died, while in most cases, the cause of death was unknown. The individual currently alive (M^e^) and the last translocated individual (G^c^) were not included in the analysis.

Of the total number of surviving specimens, 23 specimens were biopsied (15.23%) (Table 4), of which 56.5% were dead by the end of 2024. No pathogens were detected in any of the specimens analyzed (Table 4). Rapid molecular analyses of mitochondrial DNA identified all the samples analyzed as *P. nobilis*. However, the method based on nuclear DNA revealed simultaneous PCR amplification of DNA fragments from both species, indicating that some specimens were hybrids. Individuals suspected of being potential hybrids based on their morphological characteristics were confirmed as such by molecular analysis (n = 8), with 100% agreement between field assessment and genetic results, while 75% of the genetically analyzed individuals were identified as pure *P. nobilis* (Table 4). Finally, it should be noted that the sampling of the mantle tissue did not produce negative effects on the survival of individuals. In fact, biopsied individuals were monitored (longer TMA) longer than individuals that were not (Mann–Whitney U test = 149.0, *p* < 0.001) (Figure 6b). This suggests that the biopsied specimens were monitored for a longer period of time, even though this test was not exhaustive, due to the small number of observations in the biopsied group.

## 4. Discussion

Monitoring programs are essential to determine population trends or ecosystem status [82,83] and to assist the decision-making process in conservation policies. For this reason, the relevance of monitoring programs for the conservation of endangered species cannot be underestimated and their planning needs to be addressed carefully since poor designs and flawed executions frequently lead to failure [84,85]. There are factors specific to threatened species that can hinder their monitoring [86,87], such as difficulty in their detection, erratic fluctuations in populations and their scarcity [88].

The distribution of the endangered *P. nobilis* in the Mediterranean Sea has experienced a significant decrease since autumn 2016, and is currently limited to a few populations in coastal lagoons, deltas and lakes along the Mediterranean coast that seem to act as sanctuary areas [47,89,90,91,92]. In Spain, these populations can be found in areas such as the Ebro Delta and the Mar Menor lagoon [65,73,93]. The different salinity and temperature conditions compared to open water in these areas have probably hindered pathogen transmission during the MME or perhaps enhanced the health and immune system of the host [14,40,47,73,93]. However, although natural barriers effectively restricted the presence of *H. pinnae* and other pathogens in these regions, unfortunately, mortality is now expanding into areas previously considered sanctuaries [65,90,93,94,95,96,97,98].

Here we do not focus on these isolated populations, but instead, we evaluate 153 individuals of *P. nobilis* and hybrids that were found in different locations along the Spanish Mediterranean coast in open water after the onset of the MME between 2017 and 2024. These single individuals, which survived the MME, might not be able to reproduce and establish new populations, since the low density and the great distance between them hinder external fertilization, typical of marine bivalves [99,100]. Therefore, the need to understand the survival and distribution patterns of this species is paramount to implementing proper conservation measures under the current circumstances. In fact, finding living individuals of *P. nobilis* after the mass mortality arrival during this 8-year period has become a considerable challenge, specifically considering the geographical scale of the study.

Citizen science is very relevant for the monitoring of several species [101,102,103,104], since it is considered a powerful and low-cost way of monitoring critically endangered species in large geographical areas [105,106,107]. Our results confirm this, as diverse citizen science profiles provided 43.1% of the records of monitored individuals. Therefore, it is evident that collaboration between scientists and citizens is not only valuable but can also be essential when facing the challenge of monitoring a species in such a critical situation.

However, the success of such collaboration depends on the choice of the communication channels, since these differ between observer profiles. In fact, although more than half of the sightings were reported directly to the scientific team, some observers prefer to use informal methods, such as posting their findings on social media. This variation in reporting practices highlights the need for researchers to be proactive in seeking information, as not all sightings reach the scientific team directly. This has been shown before in studies where passive citizen science information (defined as any information of ecological value posted on the Internet without being linked to any specific project or campaign) has been gathered from social media to obtain records of species of interest [108,109].

A hybrid communication channel, where volunteers, like fishermen and divers, report their sightings through the citizen science platform SW, with later validation, ex situ first and in situ later, by scientific experts, accounted for only 9.2% of the surviving individuals of this study. However, during the onset of the MME, this platform provided more than 300 observations validated as *P. nobilis*, of which the majority were lost during the mortality event [73]. This shows that citizen science platforms can emerge as very useful tools during a large-scale, short-term event like an MME, for which large spatial scales need to be evaluated during a limited time period, as well as for monitoring of biodiversity and cryptic species [110,111], even though their shortcomings need to be taken into account. For instance, this includes the possible overestimation bias for benthic species [111], while it is important to provide proper training for observers and ensure expert validation, as well as offer a structured platform for reporting to improve the reliability of data [110,112].

During the monitoring period the proportion of hybrid individuals changed over time. After hybridization was first discovered in the Balearic Islands in 2017 [48] in Cabrera National Park, the proportion of living hybrids has been increasing. The first known hybrids were located in the Balearic Islands, while since 2019, there have been reports from the Spanish mainland as well. The ease of taxonomy confirmation of in situ morphological identification, through a genetic methodology [69], has been a step forward in facilitating the identification of hybrids during the monitoring surveys. Although some bivalves exhibit doubly uniparental inheritance (DUI) of mitochondrial DNA, this phenomenon has not been reported to date in *P. nobilis* [67,113]. Therefore, the presumed strictly maternal inheritance in *P. nobilis* likely accounts for the absence in our study of hybrid detection through mitochondrial analysis. In contrast, nuclear genomic data, reflecting biparental inheritance, can uncover admixture and offer a more complete understanding of hybridization events. Therefore, as the biopsies to obtain genetic data do not influence the survival of the individuals [65], this is an excellent tool to increase knowledge of the surviving individuals. In this regard, it is possible that the higher TMA observed in biopsied specimens might be attributed to the fact that longer-living specimens had a higher chance of being visited and biopsied.

Most of the specimens, regardless of the time of discovery, were found in marine protected areas, in shallow waters (in the upper 15 m) and in *P. oceanica* meadows. This distribution pattern is consistent with the conventional habitat preference [15,18,114,115]. Even after the MME, *P. oceanica* meadows still provided a suitable habitat for *P. nobilis* by providing protection from hydrodynamics [116,117], shelter from predators [32] and increased food availability by reducing current flow and trapping particles [118].

The depth of occurrence might be biased due to limitations in detecting individuals at greater depths, as shown before [89]. Deeper waters are less accessible to most citizen science profiles and require more exhaustive sampling. However, traditional ad hoc monitoring of *P. nobilis* populations prior to the MME has shown that maximum densities at Cabrera National Park ranged between 10 and 20 m depths and decreased in deeper water [20], so it is unlikely many individuals have been overlooked.

The average survival time of the individuals, estimated from the moment they were detected alive until their death (TMA), was 283.43 ± 22.51 days on average. Apart from ongoing demography monitoring of *P. nobilis* in the so-called sanctuary areas, there are only a few studies addressing the survival time of fan mussel individuals in the open sea. In Greece and Italy, individuals survived from a few months to more than a year in some cases after infection [67,94], which agrees with previous studies of individuals in captivity [47], and the findings presented in this study in the open sea.

The monitoring (survival) time in hybrid individuals was more than double that observed in *P. nobilis* specimens, suggesting that the hybrid fan mussel has a higher survival potential. These individuals may not be affected by parasites [48,67], as is the case for *P. rudis*. In fact, half of the survivors to date are hybrid individuals, causing the increase in their proportion during the years monitored. However, survival rates of fan mussels should be determined more precisely, since here the TMA was used instead of the actual survival time, which is longer. To do this, it would require knowledge of the exact age of each surviving individual, which would require resources over a spatial scale beyond the possibilities of the present study. The mortality of the monitored individuals is higher during the summer months, as previously demonstrated for the occurrence of MMEs in several Mediterranean species, which suggests a correlation between mortality and unusually high seawater temperatures, linked to a prolonged stratification of the water column in the late summer months [119,120]. These conditions lower physiological resistance to parasitic infections due to the increase in respiratory demand [121]. Therefore, the mortality peak observed after the summer could be a direct consequence of this, as, already before the MME, it has been known that *P. nobilis* incurs a period of debilitation [47,73]. The pathogen is still present in the water column [96], so the existence of surviving individuals, even years after the onset of the MME, is probably attributable to the excellent physiological conditions of these specimens, which can still survive despite the parasitic infection [47]. Although the analyzed samples did not reveal the presence of pathogens, such as *H. pinnae* and *Mycobacterium* sp., it is possible that specimens exhibiting resistance were exposed to other untested pathogens, such as Picornavirus (*Pinna nobilis* Picornavirus). This virus appears to interfere with the immunocompetence of the species and play a significant role in mass mortality [13,122].

The observation that most of the empty shells were aged <15 years when they died suggests that pathogen resistance could be age-dependent and that these resistant individuals usually have short lifespans. This observation, coupled with the absence of young recruits and juveniles, confirms the extremely dangerous alteration of the life cycle of the species, which is one of the greatest constraints for its recovery [40,41,123].

Despite the disappearance of *P. nobilis*, recent observations, supported by targeted surveys and molecular confirmation, indicate a notable expansion of *P. rudis* within the Mediterranean Sea, suggesting an ongoing ecological shift in which the species is increasingly colonizing new habitats, including areas previously occupied by *P. nobilis* [89,124,125].

Although many of the monitored individuals of *P. nobilis* were found in protected areas, the vast majority are subject to various anthropogenic pressures, with fishing activity and anchoring being the most frequent. SCUBA divers potentially represent a lesser threat to the species since diving centers do not generally target shallow meadows of *P. oceanica* [32], which is also in agreement with this research. However, it is important to note that our data do not allow us to confidently assert the contribution of these pressures to *P. nobilis* mortality since the study was not designed to explore the link between anthropogenic pressures and the causes of mortality of *P. nobilis*. However, the survival of fan mussels in the Mediterranean Sea might be favored by the regulations implemented in some Marine Protected Areas, such as no-take zones and anchoring prohibition [3].

Translocations are a valuable conservation strategy used to favor the survival of species in critical situations, such as those with strong demographic declines or a high probability of extinction [126,127,128]. The translocations during the monitoring period were carried out for these reasons and were used as a method of rescuing individuals severely threatened by the anthropogenic pressures present in their original locality. The present study, in agreement with similar research [44,45,129], did not reveal negative effects of translocations on the survival of individuals, although it is important to consider that the comparison was based on a limited number of translocations. Therefore, translocations may be considered as a management measure to be applied in a few justified cases, such as when imminent anthropological pressures cannot be promptly removed from the threatened individuals. This study provides crucial insights for the conservation management of *P. nobilis* survivors in the Mediterranean Sea, emphasizing the importance of protected areas, the potential resilience of hybrid individuals and the ongoing threats posed by anthropogenic pressures.

The spatial extension of monitoring and the combination of methodologies limited the complete recording of all the considered variables for each individual under observation. This aspect was especially reflected in the difficulty of establishing the actual date of death of the individuals. Considering the date of demise of an individual as the date when it is observed dead or not observed in the previously defined coordinates can lead to an overestimation of the time it is recorded as monitored alive (TMA). Increasing the frequency of surveys of individuals is highly recommended since this may mitigate this bias and provide a more accurate estimate of mortality.

Marine biodiversity conservation issues have become increasingly urgent due to growing anthropogenic pressure and the challenges of climate change. Therefore, it is not only important to obtain adequate knowledge of population decline, but also to translate this knowledge into effective actions to prevent species extinctions [130,131]. In particular, the current state of *P. nobilis* is particularly worrying, as no populations have been found in the open sea, but only isolated individuals and a low frequency of juvenile individuals. Therefore, it is of utmost priority to push the research towards completing the reproductive cycle of the species in captivity in order to recover populations in suitable sites, thus enhancing the natural recruitment of the species. It is also crucial to improve the understanding of the mechanisms of the disease affecting the species, which is possible, as this line of research is benefiting from the many advances in the field of genetics [132]. To do so, it is essential to continue the relentless search for surviving individuals in the open Mediterranean Sea in suitable areas such as *P. oceanica* meadows in marine protected areas, as suggested by both the results of this study and the existing literature.

## 5. Conclusions

Community participation is vital to the success of conservation achievement [130,131]. In addition to MMEs, the species faces multiple threats within its habitat. It is vital for the survival of the species to reduce these threats, especially boat anchoring, and apart from implementing conservation measures, it is essential to ensure their enforcement to actually achieve that goal. In the Balearic Islands, for example, despite the complete ban on anchoring over *P. oceanica* meadows [133], we found that 86.76% of the individuals found in this habitat are threatened to some degree by this activity.

Finally, despite the fact that more than 8 years have passed since the onset of the MME of *P. nobilis* in the Spanish Mediterranean Sea, surviving specimens are still being detected in the open sea. Regardless of the low numbers, their presence brings hope and highlights their importance in providing clues on their resistance to infection. Therefore, the conservation and protection of these survivors found in the open sea, aided by their breeding in captivity, are essential for the preservation of this emblematic species.

## Figures and Tables

**Table 1:** Number of surviving *P. nobilis* and hybrid individuals monitored each year of the monitoring period. Note that some individuals are included in more than one year if they are still alive by the end of the year. Values in square parentheses indicate the number of specimens of each taxon encountered in each year.

Year	*P. nobilis*	Hybrids	% Hybrids
2017	60	1	1.6
2018	36 [6]	1 [0]	2.9
2019	14 [8]	3 [2]	17.6
2020	21 [14]	7 [4]	25.0
2021	10 [6]	5 [0]	33.3
2022	18 [11]	8 [4]	30.8
2023	13 [2]	13 [6]	50.0
2024	8 [0]	9 [1]	52.9

**Table 2:** Age estimation of 10 empty shells of monitored survivor individuals collected during the surveys. Date expressed in day/month/year.

Collection Date	Location	Site	Species	Width	Length	Age Range (Years)
01/11/2020	Cabrera	Conejera	Hybrid	16	36	10–15
24/01/2018	Cabrera	Santa María	*P. nobilis*	17	49	7–9
01/08/2019	Cabrera	Cala Blava	*P. nobilis*	12	26	3–4
17/08/2020	Menorca	Cala Moli	*P. nobilis*	15	32	3–5
17/01/2023	Gerona	Cadaqués	*P. nobilis*	18	45	8–10
25/09/2023	Gerona	Arenella	*P. nobilis*	19	46	7–10
21/03/2023	Menorca	Es Grau	*P. nobilis*	16	37	5–7
08/11/2023	Menorca	Binisafuller	Hybrid	16	34	5–6

**Table 3:** Summary table of the translocated surviving individuals of *P. nobilis* and hybrids (2017–2024), indicating their original and recipient location. Dates of first observation, translocation and death are indicated. Letters in parentheses indicate province of the location: (C)—Cabrera, (G)—Gerona, (M)—Mallorca.

Original Location	Destination	1st Observation	Species	Translocation	Death
Es Castell (C)	Santa María (C)	26/06/2017	*P. nobilis*	07/08/2018	22/10/2018
Rodona (C) ^a^	Santa María (C)	13/06/2017	*P. nobilis*	13/06/2017	24/01/2018
Cala Blava (M)	Santa María (C)	29/01/2018	*P. nobilis*	07/06/2018	12/11/2020
Cala Blava (M)	Santa María (C)	18/12/2018	*P. nobilis*	29/04/2019	01/08/2019
Cala Blava (M) ^b^	Santa María (C)	14/02/2018	*P. nobilis*	07/06/2018	07/08/2018
Caló des Llamp (M)	Santa María (C)	23/12/2017	*P. nobilis*	07/08/2018	21/02/2019
Cala Llumenera (G) ^c^	Arenella (G)	01/09/2021	*P. nobilis*	25/09/2023	14/02/2024
Clot de la Mola (M) ^e^	Santa María (C)	10/11/2022	Hybrid	19/03/2024	-
C. San Jordi (M) ^d^	Santa María (C)	27/11/2023	Hybrid	19/03/2024	08/07/2024

^a^—individual captured by a fisherman in the nets; ^b^—sick individual; ^c^—damaged individual, bro-ken shell; ^d^—damaged individual, entangled in a fishing net or lying down at the bottom; ^e^—individual still alive at the moment of manuscript preparation.

**Table 4:** Results of molecular genetic analyses for identification of *P. nobilis* and hybrids and the detection of the main pathogens performed on samples collected from survivors (2017–2024). All samples were negative for *H. pinnae* and *Mycobacterium* sp.

Location	Site	Taxon	Multiplex PCR (nDNA)
Ibiza	Cala Espart	Hybrid	Hybrid
Cabrera	Es Castell	*P. nobilis*	*P. nobilis*
Cabrera	Cala Emboixar	Hybrid	Hybrid
Cabrera	Conejera	Hybrid	Hybrid
Cabrera	Na Pobra	*P. nobilis*	*P. nobilis*
Cabrera	Sifón Foradada	Hybrid	Hybrid
Mallorca	Cala Blava	*P. nobilis*	*P. nobilis*
Menorca	Clot de la Mola	Hybrid	Hybrid
Mallorca	Illa de Formentor	*P. nobilis*	*P. nobilis*
Menorca	Es Grau	*P. nobilis*	*P. nobilis*
Barcelona	Mataró	*P. nobilis*	*P. nobilis*
Girona	Cala Llumenera	*P. nobilis*	*P. nobilis*
Girona	Illa de S’Arenella	*P. nobilis*	*P. nobilis*
Girona	Portlligat	*P. nobilis*	*P. nobilis*
Girona	Port de la Selva	*P. nobilis*	*P. nobilis*
Girona	Illa de S’Arenella	*P. nobilis*	*P. nobilis*
Girona	Platja Can Perefet	*P. nobilis*	*P. nobilis*
Girona	Platja de Garvet	*P. nobilis*	*P. nobilis*
Girona	Badia de Colera	*P. nobilis*	*P. nobilis*
Girona	Badia de Colera	*P. nobilis*	*P. nobilis*
Alicante	Orihuela	Hybrid	Hybrid
Alicante	Moraira	Hybrid	Hybrid
Menorca	Binissafuller	Hybrid	Hybrid

## Data Availability

Affiliated institutions have a data sharing protocol; therefore, raw or processed data will be made available upon request.

## Abbreviations

The following abbreviations are used in this manuscript:

SCI	Site of Conservation Interest (European Union Nature 2000 Network)
SAC	Special Area of Conservation
DPV	Diver Propulsion Vehicle
SW	Sea Watchers (citizen science platform)
PAMS	Posterior Abductor Muscles
TMA	Time Monitored Alive
SPS	Species Protection Service of Regional government of the Balearic Islands (Spain)
DUI	Doubly Uniparental Inheritance
